# Interpolation based consensus clustering for gene expression time series

**DOI:** 10.1186/s12859-015-0541-0

**Published:** 2015-04-16

**Authors:** Tai-Yu Chiu, Ting-Chieh Hsu, Chia-Cheng Yen, Jia-Shung Wang

**Affiliations:** Department of Computer Science, National Tsing Hua University, No. 101, Section 2, Kuang-Fu Road, HsinChu, 30013 Taiwan

**Keywords:** Microarray data analyses, Gene expression, Consensus clustering, Affinity propagation, Interpolation

## Abstract

**Background:**

Unsupervised analyses such as clustering are the essential tools required to interpret time-series expression data from microarrays. Several clustering algorithms have been developed to analyze gene expression data. Early methods such as k-means, hierarchical clustering, and self-organizing maps are popular for their simplicity. However, because of noise and uncertainty of measurement, these common algorithms have low accuracy. Moreover, because gene expression is a temporal process, the relationship between successive time points should be considered in the analyses. In addition, biological processes are generally continuous; therefore, the datasets collected from time series experiments are often found to have an insufficient number of data points and, as a result, compensation for missing data can also be an issue.

**Results:**

An affinity propagation-based clustering algorithm for time-series gene expression data is proposed. The algorithm explores the relationship between genes using a sliding-window mechanism to extract a large number of features. In addition, the time-course datasets are resampled with spline interpolation to predict the unobserved values. Finally, a consensus process is applied to enhance the robustness of the method. Some real gene expression datasets were analyzed to demonstrate the accuracy and efficiency of the algorithm.

**Conclusion:**

The proposed algorithm has benefitted from the use of cubic B-splines interpolation, sliding-window, affinity propagation, gene relativity graph, and a consensus process, and, as a result, provides both appropriate and effective clustering of time-series gene expression data. The proposed method was tested with gene expression data from the Yeast galactose dataset, the Yeast cell-cycle dataset (Y5), and the Yeast sporulation dataset, and the results illustrated the relationships between the expressed genes, which may give some insights into the biological processes involved.

## Background

In the last two decades, the development of medicine and molecular biology has been significantly improved by DNA microarray technology applications. The technology allows variations in expression levels to be monitored simultaneously for thousands of genes, even in some multiple experiments in which data are collected across various time points. During distinct biological processes, high-throughput data of time-series gene expression are recorded to explore the complex dynamics of biological systems. The expression data can reveal gene activities in conditional reactions such as cell-cycle, disease progression, and response to external stimuli (e.g., drugs and stress).

Analyses of microarray data are essential in several time-series expression experiments such as biological systems, infectious diseases, and genetic interactions [1]. Pattern recognition techniques are helpful [2] to explore and exploit high-throughput screening data from microarrays. By using these techniques, similar expression patterns can be organized into a group. In addition, phenotypic responses triggered by the production of proteins coded by the expressed mRNAs are assumed to have a causal relationship with the gene expression [3]. Therefore, numerous efficient clustering algorithms have been developed to analyze gene expression data. Some of the older methods such as k-means, hierarchical clustering, and self-organizing maps [4] are popular for their simplicity. However, the accuracy of these common algorithms is low because of background noise caused by experimental errors and uncertainty of measurement. Gene expression is a temporal process, therefore the relationship between successive time points should be considered. Some algorithms have been designed for time-series clustering, which involves temporal dependency as a critical factor. Model-based clustering methods, for example, use statistical and probabilistic models to determine the characteristics of data ([5-9]). The main advantage of model-based methods is their tolerance toward experimental errors, including noises and missing values from measurement. Model-based approaches are based on probabilistic models rather than raw expression values to maximize the likelihood of time-series expression data. However, model-based clustering methods require profitable pre-training and, in particular, suffer from computation inefficiency while modeling the gene expression profiles for clustering. In addition, considering that a biological process is a continuous function, the datasets collected from experiments are often found to contain an insufficient number of data points. Therefore, generating these missing data is another problem that researchers need to address [10]. To solve this problem, we used cubic B-splines [11] interpolation to compensate for missing data in time-course datasets, which may help disclose some unobserved details.

Affinity propagation [12] is an effective unsupervised clustering scheme. Based on this concept, with some restrictions relaxed, a method called soft-constraint affinity propagation (SCAP) [13] has been proposed for use in gene expression clustering.

In this paper, we combined the cubic B-splines interpolation, affinity propagation, and consensus clustering ([14-17]) to analyze the dependency on various time intervals between genes. Using a sliding-window mechanism, clustering results obtained from affinity propagation were merged for final clustering partition with consensus clustering.

## Methods

**Figure 1:**
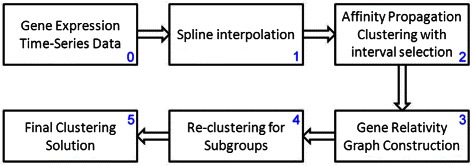
Framework of the proposed affinity propagation-based clustering algorithm with 6 steps.

Here, the proposed algorithm based on B-splines interpolation [10], affinity propagation [12], and consensus clustering [14] is described. The time-course gene expression clustering problem was formulated as follows: for a set of genes *G*={*G*_1_,*G*_2_,…,*G*_*n*_} where *n* is the number of genes, and each gene *G*_*i*_ includes *τ* time points for the gene expression values, the *n* genes are grouped into *K* disjoint clusters *C*_1_,*C*_2_,…,*C*_*K*_. Based on the clustering, various groups of genes with similar expressions can be identified and organized for further analyses. The framework of the proposed algorithm is shown in Figure 1. First, the original *τ* time points are doubled to give 2*τ*−1 points using the cubic B-splines interpolation algorithm [11]. Second, a sliding-window mechanism is applied to extract the possible features within the extended time-course gene expression profiles. Then, affinity propagation is performed to form the gene cluster. Third, a voting mechanism for gene grouping is applied and construction of the gene-relativity graph is performed. Noted that Steps 2-3 are corresponding to the construction of the gene relatively graph, which is the time consuming part. Once the graph is settled, we generate 31 grouping results corresponding to 31 different thresholds *σ* = 0.50, 0.51, …, 0.80. In Step 4, each of these grouping results is fine-tuned by the re-clustering algorithm to reduce the size of clusters. Step 3 evaluates the SI (Silhouette Index) of each grouping and output the one with the largest SI value finally.

### Spline interpolation

**Figure 2:**
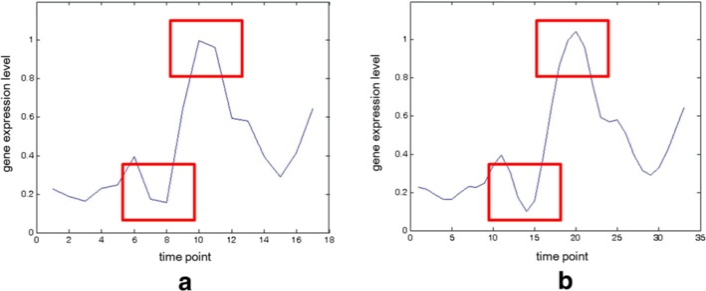
An example.**(a)** The original gene data. **(b)** The corresponding spline-interpolated gene data.

Cubic B-splines were used to represent gene expression curves to obtain a continuous time formulation. After spline interpolating, the curve can be resampled to estimate expression values at any time point; for example, for the case shown in Figure 2, the spline-interpolated gene data are smoother than the original data. However, too many resample points may cause fallacy and inefficiency. In the proposed algorithm, one point in each time interval is resampled, which doubles the number of expression data points to 2*τ*−1.

### Affinity propagation clustering with interval selection

Affinity propagation [12] was proposed by Frey and Dueck in the spirit of massage passing which is approximated following the concepts of belief-propagation [18]. It chooses the viewpoint of all data points could be exemplars potentially, and takes the measure similarity between pairs of data points as input. Besides, the preference of each data point has also been taken as input to decide whether a data point should be chosen as an exemplar for larger value of preference. The preference can be used to control the number of clusters and is suggested to be the median of the input similarities for a moderate number of clusters.

The traditional clustering algorithm, such as k-centers clustering, generally begins with a set of randomly selected exemplars (i.e., centroid in clusters) and minimizes the error function for convergence iteratively. Compared to k-centers clustering, affinity propagation avoids the drawback of improper exemplars initialization which is far to a correct solution.

To take the temporal relationship and interval between time points into account, we used a sliding-window mechanism to evaluate all sub-intervals as features. It should be noted here that, unlike pattern recognition in image processing where the feature selection is employed for dimensionality reduction because of the rich information available in an image, time-courses in gene expression are relatively small. Therefore, we applied the sliding-window mechanism for informative feature selection so that all possible segments could be explored.

Affinity propagation clustering [12] is self-organized without the need to input the anticipative number of clusters *K*. Based on this, affinity propagation was applied to group genes in each window while discovering the relationship between genes in different time intervals in unsupervised mode.

**Figure 3:**
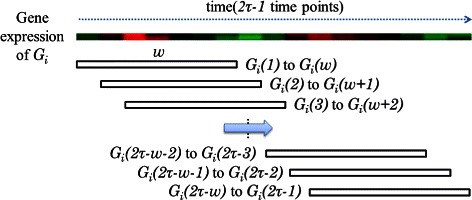
Sliding-window mechanism for interval selection.

For each gene expression of *G*_*i*_ with 2*τ*−1 time points, the sliding-window mechanism with size *w* first generates a series of time patterns, (*G*_*i*_(*t*) to *G*_*i*_(*t*+*w*−1))s, as depicted in Figure 3. A total of 2*τ*−*w* time patterns are obtained for each gene.

To measure the similarity of two genes *G*_*i*_ and *G*_*j*_, the feature *C* *o* *r* *r*(*G*_*i*_,*G*_*j*_), which corresponds to the subpattern (*G*_*i*_(*t*) to *G*_*i*_(*t*+*w*−1)), is calculated by the Pearson correlation coefficient as:

(1) $$\begin{array}{@{}rcl@{}} Corr(G_{i},G_{j})&=& \\ && \frac{\sum_{l=t}^{t+w-1}(G_{i}(l)-\overline{G_{i}})(G_{j}(l)-\overline{G_{j}})} {\sqrt{\sum_{l=t}^{t+w-1}\left(G_{i}(l)-\overline{G_{i}}\right)^{2}\sum_{l=t}^{t+w-1}\left(G_{j}(l)-\overline{G_{j}}\right)^{2}}} \end{array} $$

where *G*_*i*_(*l*) and *G*_*j*_(*l*) are the expression values at the *l*th time point of *G*_*i*_ and *G*_*j*_, respectively, and $\overline {G_{i}}$ and $\overline {G_{j}}$ are the mean values of *w* expression data of the two genes.

Let ***CM*** be the correlation matrix, where entry *r*_*ij*_ is the similarity *Corr*(*G*_*i*_,*G*_*j*_). As suggested in [12], we choose the median of those similarities as the *preference* value for affinity propagation input. For each gene *G*_*i*_, affinity propagation is applied to assign a categorized label *y*_*i*_ as the most similar exemplar of *G*_*i*_. After the affinity propagation clustering is completed, the categorized label for each gene can be obtained. Accordingly, an adjacency matrix ***M*** is formed with an entry *m*_*ij*_ defined as:

(2) $$\begin{array}{@{}rcl@{}} m_{ij}= \left\{ \begin{array}{ll} 1 & \text{if}\; y_{i} = y_{j}\\ 0 & \text{if}\; y_{i} \neq y_{j} \end{array} \right. \end{array} $$

where *y*_*i*_ and *y*_*j*_ are the labels of the genes *G*_*i*_ and *G*_*j*_, respectively.

### Gene-relativity graph construction

As mentioned, there are (2*τ*−*w*) windows; thus, (2*τ*−*w*) adjacency matrices *M*_1_,*M*_2_,…,*M*_2*τ*−*w*_, will be constructed to represent the temporal dynamics between genes in the sliding-window mechanism. Next, the (2*τ*−*w*) adjacency matrices are summarized into one consensus matrix ${M_{w}^{c}}$ by simply merging as:

(3) $$ {M_{w}^{c}}=\frac{1}{2\tau - w}\sum_{u=1}^{2\tau - w}M_{u}  $$

where the entry *m*_*ij*_ in the consensus matrix ${M_{w}^{c}}$ indicates the possibility that the genes *G*_*i*_ and *G*_*j*_ are in the same class.

The window size *w* can be changed to further observe the relationship between genes. For each *w*, one consensus matrix is constructed for each window size, so, for *l* window sizes *w*_1_, *w*_2_, …, *w*_*l*_, *l* corresponding consensus matrices $M_{w_{1}}^{c}$, $M_{w_{2}}^{c}$, …, $M_{w_{l}}^{c}$ are obtained. We also defined an aggregated consensus matrix *R* as:

(4) $$ R=\frac{1}{l}\sum_{u=1}^{l}M_{w_{u}}^{c}  $$

where each entry in the aggregated consensus matrix *R* denotes the probability of two genes, *G*_*i*_ and *G*_*j*_, appearing in the same class.

Afterwards, a graph is constructed to represent the relationship between genes from the aggregated consensus matrix *R*, called the gene-relativity graph *P*=(*G*,*R*). The vertices of the gene-relativity graph correspond to the genes in *G*, and the edges indicate the probability that two genes will eventually appear in the same class.

### Graph partitioning for class discovery

The gene-relativity graph *P* can be used to investigate the relationship between genes. A relativity threshold *σ*≥0.5 was chosen to convert the graph *P* into a binary graph *P* ^*b*^:

(5) $$\begin{array}{@{}rcl@{}} p_{ij}^{b}= \left\{ \begin{array}{ll} 1 & \text{if}\; p_{ij} \geq \sigma\\ 0 & \text{if}\; p_{ij} < \sigma \end{array}\right. \end{array} $$

where *p*_*ij*_ and $p_{\textit {ij}}^{b}$ are the edge weights between genes *G*_*i*_ and *G*_*j*_ of *P* and *P* ^*b*^, respectively.

Next, a depth-first search algorithm was employed to find connected components of the undirected graph *P* ^*b*^. Suppose *L* connected components *C*_1_,*C*_2_,…,*C*_*L*_, are found, then these *L* connected components could be considered to be the *L* disjoint clusters *C*_1_,*C*_2_,…,*C*_*L*_. However, some edges weighing more than 0.5 but slightly less than the relativity threshold *σ* in the gene-relativity graph *P* are eliminated from the binary graph *P* ^*b*^, which produces several connected components (i.e., clusters) with a few vertices (i.e., genes) when the depth-first search algorithm is applied. In our experience, this is a common problem in processing affinity propagation results. Connected components with a few vertices, called *sub-clusters*, can influence the clustering results, so, in our algorithm, the current clustering results are rearranged using a refinement process to reduce the influence of noise.

To determine which cluster is minor (sub-cluster), a threshold parameter *φ* was used to restrict the number of genes in one cluster. That is, a cluster is denoted as *sub-cluster* if the number of genes in the cluster is less than *φ*. For each cluster *C*_*p*_, 1≤*p*≤*L*, the number of genes is compared with the parameter *φ* to identify *L* ^′^ *sub-clusters* with $n_{1}, n_{2}, \ldots, n_{L^{\prime }}\phantom {\dot {i}\!}$ number of genes in each.

Next, the refinement process is applied by merging each *L* ^′^ minor *sub-clusters* with *L*−*L* ^′^ major clusters with more than *φ* genes.

Consider each *sub-clusters* *C*_*q*_ with the *n*_*q*_ number of genes. If *n*_*q*_ is equal to one, this is a singleton cluster with one gene, say *G*_*q*_. Our algorithm first identifies the gene *G*_*h*_ that has the highest relativity to *G*_*q*_ in the gene-relativity graph *P*. Then, *C*_*q*_ is processed using the following rule:

**Rule 1:**

(1.1) If the relativity between *G*_*q*_ and *G*_*h*_ is below 0.5, the merging process stops and *C*_*q*_ survives as a singleton cluster.
(1.2) If *C*_*h*_ is a major cluster, *C*_*q*_ is merged into *C*_*h*_.
(1.3) Otherwise (*C*_*h*_ is a sub-cluster), *C*_*q*_ and *C*_*h*_ are merged to form a new cluster with *n*_*q*_+*n*_*h*_ number of genes; and the type, *sub-cluster* or major, is checked.

When *C*_*q*_ is not a singleton, the algorithm calculates the mean values ${G_{q}^{m}}$ of these *n*_*q*_ gene profiles. Then, the maximum Pearson correlation coefficient is compared with the mean value ${G_{h}^{m}}$, which belongs to *C*_*q*_ in all major clusters. Next, *C*_*q*_ is merged using the following rule:

**Rule 2:**

(2.1) If the Pearson correlation coefficient between ${G_{q}^{m}}$ and ${G_{h}^{m}}$ is below 0, the merging process is stopped.
(2.2) Otherwise, *C*_*q*_ and *C*_*h*_ are merged.

The above rules are performed repeatedly until no *sub-cluster* exists and, finally, the *K* number of disjoint clusters *C*_1_,*C*_2_,…,*C*_*K*_ are obtained.

The proposed algorithm is listed below.



### The computational complexity of the proposed algorithm

The main idea of our proposed clustering algorithm is to collect enough consensus votes so as to estimate the matching probability between each pair of genes. So we construct the gene relativity graph in Step 2. Within this step, the affinity propagation procedure will be called several times, normally the number is $\sum _{w=l}^{u}(2w-\tau)$, where $l=\lfloor \frac {2\tau - 1}{2}\rfloor $ and $u = \lfloor \frac {3(2\tau - 1)}{4}\rfloor $. Since the AP algorithm has a quadratic complexity [19]. Thus, the computational complexity of Step 2 is *O*(*n* ^2^)*O*(*τ* ^2^), where *n* is the number of genes and *τ* is the number of time points. Consider Step 3. The kernel is to discover the partition (the set of connected components) using the depth-first search for each threshold, thus the computational complexity of Step 3 is 31×*O*(|*E*|), where |*E*| is the number of edges in the relativity graph. Since |*E*|=*O*(*n* ^2^), the computational complexity of Step 3 is *O*(*n* ^2^). Notice that, the optimal relativity threshold can be located (Step 3-3) with the help of the Silhouette index. As for the refinement step (Step 4), we iteratively apply both the Rules 1 and 2 to merge each of small-size clusters with some of the major clusters. Let *c* denote the number of connected components. In each iteration we need *O*(*c* ^2^) for merging. Thus, the computational complexity of Step 4 is *O*(*c* ^2^) because the number of iterations is 500 in our settings. Therefore, the overall computational complexity is *O*(*n* ^2^ *τ* ^2^)+*O*(*c* ^2^), where *n* is the number of genes, *τ* is the number of time points, and *c* is the number of initial clusters. Notice that, the number *c* is much small than *n* generally.

### Extended version of the proposed algorithm

As mentioned, in some datasets, the collection of data points is insufficient. To alleviate the possible unsatisfactory performance of the proposed algorithm because of the lack of information of short time-series (*τ*<10), an extended version of our algorithm was proposed.

Recall that the degree of investigation of the relationship between genes is influenced by the window size *w* and the number of time-points *τ* because the number of times affinity propagation clustering is repeated is decided by *w* and *τ*.

**Figure 4:**
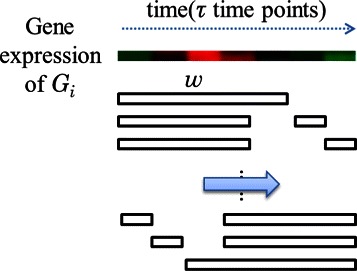
Extended version of the sliding-window mechanism for interval selection.

For *τ*(*τ*<10) time points, the window size is *w* and the number of times for affinity propagation would be (2*τ*−*w*). To increase the degree of investigation of the relationships between genes, combinations $C_{w}^{\tau }$ of sub-features selection were suggested. That is, *w* time points are chosen randomly from *τ* time points as feature vectors for the affinity propagation clustering. As depicted in Figure 4, the feature is a subset instead of a segment. Because $C_{w}^{\tau }$ is more than (2*τ* − *w*), the relationships between genes are more precise and clustering accuracy is enhanced. However, the efficiency of the algorithm will decrease because extra affinity propagations are performed. Therefore, we strongly suggest that the extended version of the algorithm is executed only when analyzing short time-series datasets (*τ*<10).

### Prediction of optimal threshold values

As discussed before, Steps 2-3 are corresponding to the construction of the gene relatively graph *P*=(*G*,*R*). The vertices of the graph correspond to the genes in *G*, and the edges indicate the probability that two genes may appear in the same class. And for each relativity threshold *σ*, a depth-first search algorithm was employed to find connected components, which correspond to the initial clustering for *σ*. Thus, we adopt these 31 initial clusters to predict the optimal threshold value using the Silhouette index. And Step 4 (re-clustering) is excluded in the determination because it is unstable in our experience. We mainly trust on the probability matrix *R*.

## Results and discussions

The adjusted Rand index and the Silhouette index are adopted to judge the clustering accuracy of our algorithm are described. Then, several real gene expression datasets were used to evaluate the proposed algorithm. Finally, the parameter settings and the performance evaluation are discussed.

### Measure of agreement

#### Adjusted Rand index

The adjusted Rand index (Hubert and Arabie [20]; Yeung and Ruzzo [21]) is a popular similarity measure of agreement between two partitions against external criteria. Using this validity index, the partition *U*={*u*_1_,*u*_2_,…,*u*_*R*_} given by the clustering result and *V*={*v*_1_,*v*_2_,…,*v*_*C*_} a *priori* classification can be compared. The adjusted Rand index (ARI) is defined as:

(6) $$ ARI=\frac{\sum_{i=1}^{R}\sum_{j=1}^{C}\binom{n_{ij}}{2} - \frac{\sum_{i=1}^{R}\binom{n_{i*}}{2}\sum_{j=1}^{C}\binom{n_{*j}}{2}}{\binom{n}{2}}}{\frac{\sum_{i=1}^{R}\binom{n_{i*}}{2} + \sum_{j=1}^{C}\binom{n_{*j}}{2}}{2} - \frac{\sum_{i=1}^{R}\binom{n_{i*}}{2}\sum_{j=1}^{C}\binom{n_{*j}}{2}}{\binom{n}{2}}}  $$

where *n*_*ij*_ is the number of objects in clusters *u*_*i*_ and *v*_*j*_, and *n*_*i*∗_ and *n*_∗*j*_ are the number of objects in clusters *u*_*i*_ and *v*_*j*_, respectively. From this definition, ARI gives the value [0, 1] to assess the degree of agreement. High values indicate that *U* is more similar to *V*; in particular, the value 1 indicates absolute agreement between the partitions *U* and *V*.

#### Sensitivity, specificity, Jaccard index and Minkowski measure

Schliep et al. [7] suggested the use of sensitivity and specificity as complementary to the use of ARI. Let *TP* denote the number of pairs in the same cluster in *U* and same priori classification class in *V*; *FP* denote the number of pairs in the same cluster in *U* and distinct class in *V*; *FN* denote distinct cluster in *U* and same class in *V*, *TN* denote distinct cluster in *U* and distinct class in *V*. The index sensitivity is defined as $\frac {\#TP}{\#TP + \#FN}$ and specificity defined as $\frac {\#TP}{\#TP + \#FP}$.

The Jaccard index *JC* is defined as $\frac {\#TP}{\#TP + \#FN + \#FP}$, another agreement between *U* and *V*, while, Minkowski measure illustrates the proportion of disagreements to *#* *T* *P*+*#* *F* *N*. Noted that all indices except ARI (or RI), do not involve the term *TN*, because this term would dominate the other three terms in both good and bad solutions [22].

(7) $$ Minkowski \, measure={\sqrt{\frac{\#FP + \#FN}{\#TP + \#FN}}}  $$

#### Silhouette, Dunn’s and Davis-Bouldin index

The Silhouette index (Rousseeuw [23]) is an internal cluster validity index that is used when true class labels are unknown. With a clustering solution *C*, the Silhouette index is used to judge the quality and to determine the proper number of clusters within a dataset. For each data point *i*, the Silhouette index is defined as:

(8) $$ s(i)=\frac{b(i) - a(i)}{\max(a(i), b(i))}  $$

where *a*(*i*) is the average dissimilarity of *i* with other data points in the same cluster, and *b*(*i*) is the minimum average dissimilarity of *i* with other data points in other clusters. The Silhouette index *s*(*C*) is the average of the Silhouette width for all data points the value [-1, 1] reflects how appropriately the data has been clustered. A high Silhouette index indicates a good clustering result, which indicates the data are classified appropriately.

The Dunn’s validation index (*DI*) is defined as

(9) $$ DI=\min_{1\leq i\leq R}\left\{\min_{1\leq j\leq R, j \neq i}\left\{\frac{\delta(u_{i}, u_{j})}{\max_{1\leq k\leq R}\{\Delta(u_{k})\}}\right\}\right\}  $$

where *δ*(*u*_*i*_,*u*_*j*_) defines the distance between clusters *u*_*i*_ and *u*_*j*_ (inter-cluster distance); *Δ*(*u*_*k*_) represents the intra-cluster distance of cluster *u*_*k*_, and *R* is the number of clusters of partition *U*. Likewise, the large values *DI* correspond to good clusters.

The Davies-Bouldin index (DBI) is defined as

(10) $$ DBI=\frac{1}{R}\sum_{i=1}^{R}\max_{i\neq j}\left\{\frac{\Delta(u_{i}) + \Delta(u_{j})}{\delta(u_{i}, u_{j})}\right\}  $$

Unlike *SI* and *DI*, inter-cluster distance *δ*(*u*_*i*_,*u*_*j*_) is in the denominator [24], small values of *DBI* correspond to compactness, thus, the cluster configuration that minimizes *DBI* indicates a good clustering result.

Besides, some recent and effective measures ([25-27]) of the reliability of the clusters can be used to evaluate the results as well.

### Time-series datasets

#### Yeast galactose dataset

The Yeast galactose dataset (Ideker et al. [28]) was built to study integrated genomic data and is composed of gene expression measurements for 205 genes involved in galactose use in *Saccharomyces cerevisiae*. The gene expression profiles were measured with four replicate assays across 20 time points (20 perturbations in the galactose pathway) and the genes have been annotated in four functional categories in the Gene Ontology (Ashburner et al. [29]) listings. For external validation, we chose the mean of four replicates at each time point to judge the clustering results against the four published functional categories.

#### Yeast cell-cycle dataset

**Figure 5:**
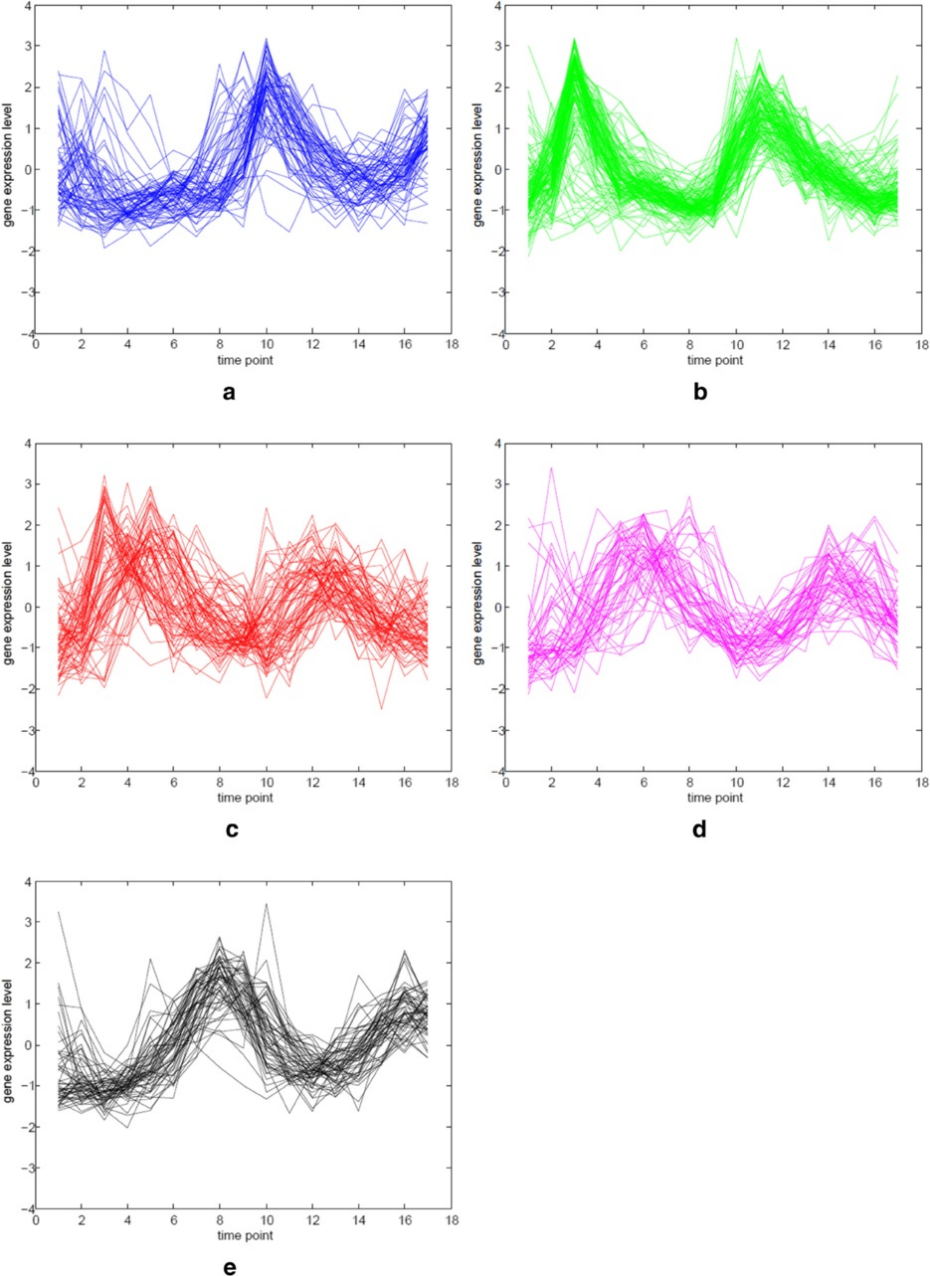
Gene expression profiles for the five phases of Y5.**(a)** Early G1 (G1E) phase. **(b)** Late G1 (G1L) phase. **(c)** S phase. **(d)** G2 phase. **(e)** M phase.

**Figure 6:**
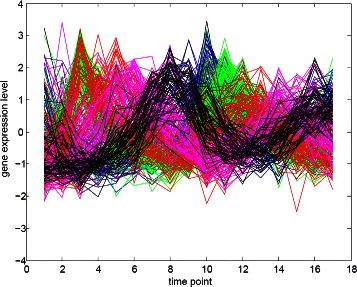
Combined gene expression profiles of Y5. The colors represent the same phases as those listed in this figure.

The Yeast cell-cycle dataset (Cho et al. [30]) includes more than 6000 yeast genes and their expression levels measured during two cell cycles at 17 time points. Schliep et al. [7] used a subset of the Yeast cell-cycle dataset, called Y5, and identified the peak time points of 17 time points as the 5-phase of the cell-cycle. The Y5 dataset consists of 384 genes that are all annotated with five phases: early G1 (G1E), late G1 (G1L), S, G2, and M. We used the standardized expression levels to enhance the performance of our algorithm and compared the results with other studies. Figure 5 and Figure 6 represent the gene expression profiles for the Yeast cell-cycle dataset (Y5). Different peak time points can be seen for the five annotated phases in Figure 5.

#### The Yeast sporulation dataset

**Figure 7:**
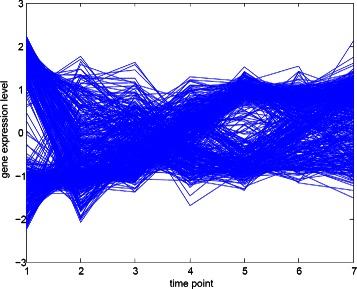
Combined gene expression profiles of the Yeast sporulation dataset.

The Yeast sporulation dataset (Chu et al. [31]) contains the expression levels of more than 6000 genes measured during the sporulation process of budding yeast across seven time points (0, 0.5, 2, 5, 7, 9, and 11 h). Genes with missing expression values and genes that showed no significant changes in expression during the process were excluded from the experimental analyses (Bandyopadhyay et al. [32]). The final dataset consisted of 474 genes for which no annotation could be assigned. Figure 7 represents the gene expression profiles for the Yeast sporulation dataset.

### Parameter settings

Here, we discuss the parameter settings used in our algorithm for the experimental evaluation. The similarities *s* for affinity propagation were chosen as the similarities between gene expression profiles based on the Pearson correlation. The preferences *p* were chosen as the medians of the similarities *s*. In addition, the window size *w* and the number of windows *l* are tradeoff between clustering accuracy and algorithm efficiency. Smaller window sizes could miss the dynamic of the temporal gene expression while larger ones could decrease the number of votes, making the relationships between genes difficult to determine. The parameter relativity threshold *σ* for graph partitioning ranged from 0.5 to 0.8 in the experimental evaluation.

### Choosing proper window sizes

In our implementation we prefer to apply a large amount of windows (*O*(*τ* ^2^), *τ* is the number of points) to collect enough consensus votes to reach the precise probability between each pair of genes. Thus, we recommend that the proper window sizes *w* range from $\lfloor \frac {2\tau - 1}{2}\rfloor $ to $\lfloor \frac {3(2\tau - 1)}{4}\rfloor $ where *τ* is the number of time points for the gene expression values being analyzed, and the appropriate number of windows *l* is more than or equal to $\lceil \frac {2\tau - 1}{4}\rceil $. Please note that the small window size might not yield a suitable clustering because insufficient information may let the affinity propagation procedure perform unstable (that is, large variation of the number of clusters). Thus, some bad votes will be included in the aggregated consensus matrix. In our experience, especially in the case of yeast sporulation, expanding the window size is necessary.

### Experimental results

For datasets which have been annotated, the clustering results are compared with the adjusted Rand index, specificity and sensitivity; otherwise the Silhouette index and Davies-Bouldin index are used for validation. As mentioned before, in the implementation we adopt 31 partitions to predict the optimal threshold value using the Silhouette index. Our program are coded in MATLAB, been running on Intel Core-i7(3.33 GHz) with 8GB memory using Windows 7 64-bit.

#### The Yeast galactose dataset

**Figure 8:**
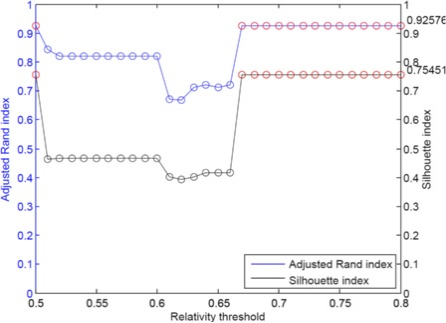
The clustering performance for the Yeast galactose dataset.

Consider Figure 8. As the indication in the curve of SI, our algorithm has a maximum value of the adjusted Rand index of 0.92576 with relativity threshold 0.67 (the leftmost point with SI value 0.75451). All of the results show that the true number of clusters are captured as the four clusters in the dataset which is annotated as four GO categories. Please note that we do not perform the spline interpolation because this dataset is simple. And the range of window size is set as (10, 15), our regular setting.

We compare our results with other methods in the literatures as shown in Table 1. Four experimental evaluation, including CRF [9], CORE [33], Ng et al. [8], and Yeung et al. [5]. This dataset is quite simple thus most of the algorithms perform well, ARI > 0.925.

**Table 1:** **The comparison of performance for the Yeast galactose dataset**

**Method**	**The adjusted rand index**
Our algorithm	0.92576
Ng *et al.*’s (2006) [8]	0.9780
Yeung *et al.*’s (2003) [5]	0.9680
CRF (Li *et al.*, 2008) [9]	0.9478
CORE (Tjaden, 2006) [33]	∼ 0.7

#### The Yeast cell-cycle dataset (Y5)

**Figure 9:**
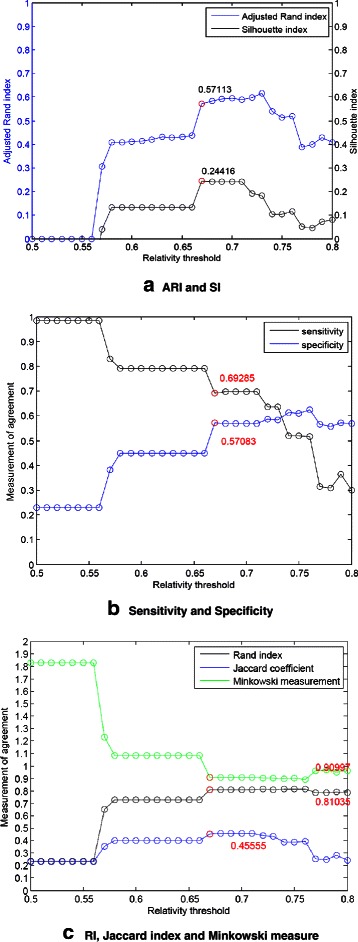
The clustering performance for Y5.**(a)** ARI and SI. **(b)** Sensitivity and Specificity. **(c)** RI, Jaccard index and Minkowski measure.

In Figure 9(a), the ARI and SI plots are varying significantly even if we have built the aggregated consensus matrix of probabilities of pairs of two genes being in the same group through the collection of enough votes. Only two thresholds 0.67 and 0.68 let the ARI of value 0.60789. So, for this tough case, our algorithm exhaustively checks 31 partitions (corresponding to 31 thresholds, 0.50, 0.51, …, 0.80) to locate the threshold with the help of SI. Here the largest SI value is 0.24416, thus we have the corresponding ARI value 0.57113. After interpolation with 33 time points, the range of window size is set as (16, 24), our regular setting.

In Figure 9(b) and 9(c), the other five measurements: sensitivity, specificity, Rand, Jaccard and Minkowski are depicted.

Compare with the results of other methods without partial learning of labeled data including k-means, cubic splines [10], different HMM model [7], CRF [9], and Chiu et al. [34]. The contrasts between these methods are listed in Table 2.

**Table 2:** **The comparison of performance for the Yeast cell-cycle dataset (Y5)**

**Method**	**ARI**	**Specificity**	**Sensitivity**
Our Algorithm	0.57113	0.69285	0.57083
Chiu et al. [34]	0.51290	0.60372	0.66061
CRF [9]	0.48080		
HMM [7]	0.46700	0.559	0.684
K-means	0.43000	0.563	0.557
Splines [10]	0.36200	0.494	0.516

Consider Table 2. The performance of our algorithm is superior to k-means algorithm which needs the number of clusters as parameter for input, and also outperforms the Splines model which using spline curves to represent gene expression profiles [10] and our early works (Chiu et al. [34]). Furthermore, the performances of probabilistic sequence data models such as HMM (Hidden Markov models) [7] and CRF (conditional random fields) [9] are also inferior to ours. We also applied six different ranges of window size, (15, 23), (16, 24), …, and (20, 28) to demonstrate the average performance. The corresponding ARI values (fixed by SI values as discussed in Figure 9(a)) are 0.58330, 0.57113, 0.52061, 0.51881, 0.40816 and 0.53399, the average value is 0.52267.

**Figure 10:**
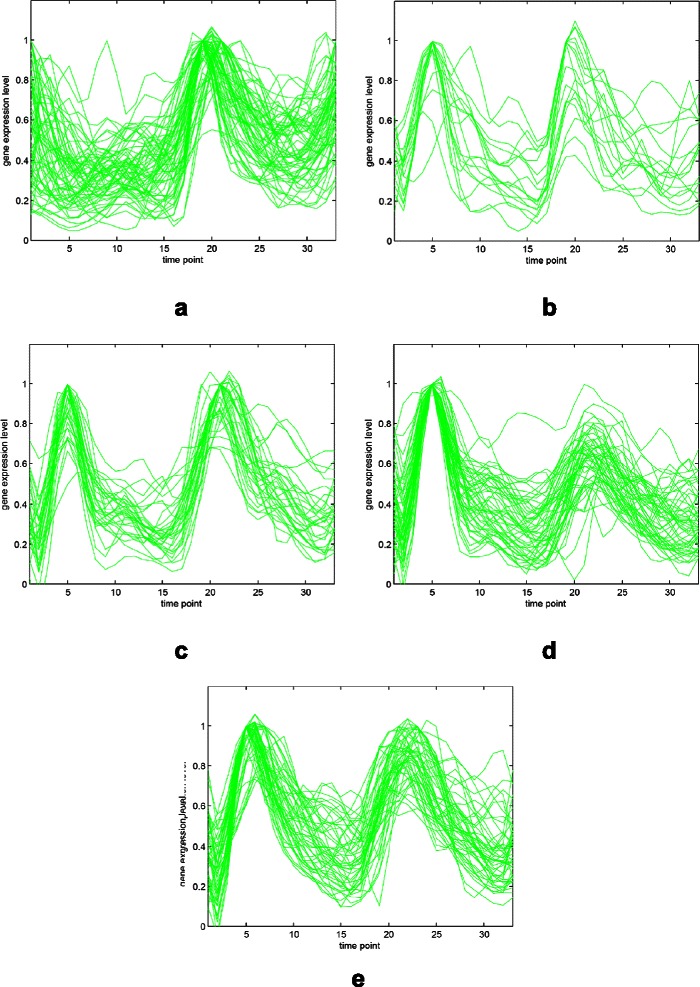
The gene expression results for Y5.**(a)-(e)** are five resulting classes categorized from our algorithm.

Even though, in contrast to the results derived from the Yeast galactose dataset, our best result of 0.57113 of the adjusted Rand index is not looking good. To take a closer look on the phenomenon, we demonstrate the clustering result with five groups derived from our algorithm in Figure 10. Due to the ambiguities of the peak time points as annotation among the five groups in the Yeast cell-cycle dataset (Y5), the clustering result can not be improved to higher values. Besides, the outliers and noises of the dataset also impact on the clustering accuracy even if our algorithm applies voting mechanism for diminishing the influence of noises.

**Figure 11:**
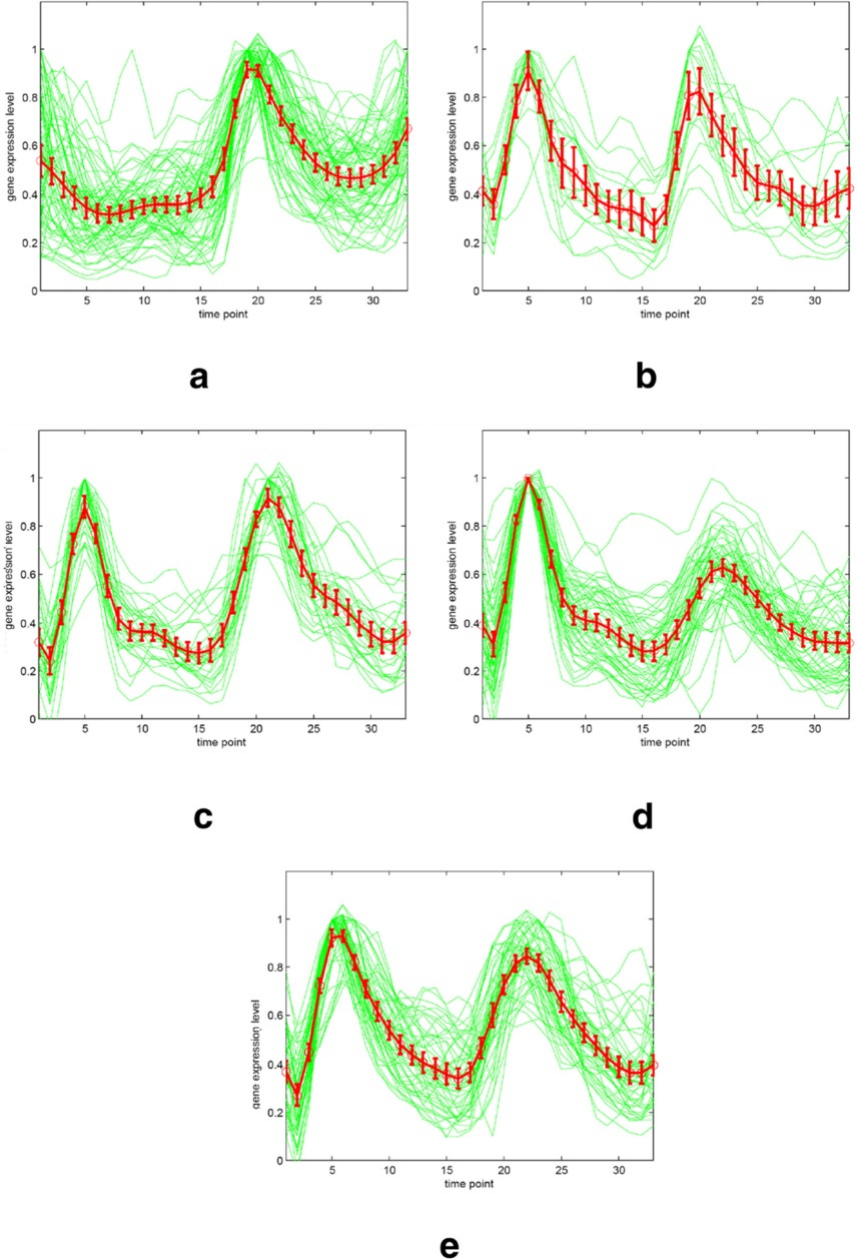
The gene expression results for Y5 with 33 time points (interpolated).**(a)-(e)** The corresponding five resulting classes categorized from our algorithm.

**Figure 12:**
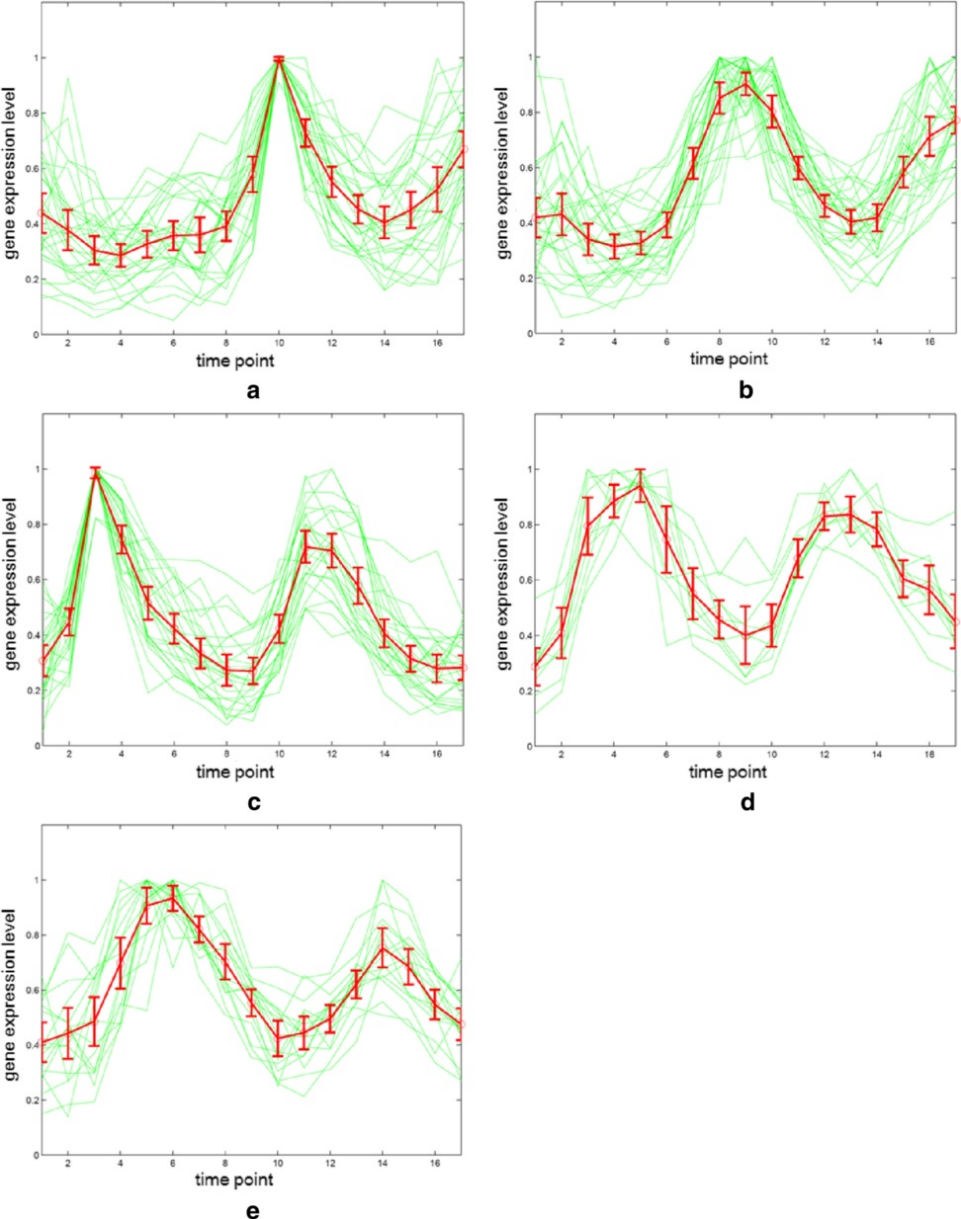
The gene expression results for Y5 with original 17 time points.**(a)-(e)** The corresponding five resulting classes categorized from our algorithm.

The relationship between normalized gene expression values of the genes and the time points for each cluster are indicated by cluster profile plots. The best clustering results of cluster profile plots evaluated by our proposed algorithm are shown in Figure 11 and Figure 12 (green). In Figure 11, the clusters with 33 time points are extracted from original Yeast cell-cycle dataset (Y5) by using the spline interpolation. In addition, the original 17 time points of Y5 are depicted in Figure 12.

The clustering performance can be observed by the gene expression of each cluster shown in Figure 11 and Figure 12 from (a) to (e). The first (a), second (b) and fifth (e) clusters are almost perfectly separated. The mean values and the confidence intervals (95%) are plotted as red lines.

#### The Yeast sporulation dataset

**Figure 13:**
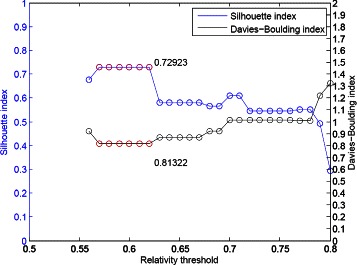
The clustering performance for the Yeast sporulation dataset with spline interpolated data (13 time points).

Due to the lack of annotations on the Yeast sporulation dataset, we use two internal validity measurements, i.e., the *SI* and *DBI* to judge the clustering performance. Amongst all 31 relativity thresholds, our algorithm has a maximum value of the Silhouette index of 0.72923 as shown in Figure 13. To take a closer look at the results, a lower *σ* causes distinct groups combined into a larger group including all 474 genes. The effect is caused by the insufficient number of time points which impacts the number of votes for investigating the relationships between genes. Noted that there are only seven time points on the Yeast sporulation dataset. As discussed in the window-size setting, for this Yeast sporulation dataset, we have to expand the window size to prevent some bad votes been included in the aggregated consensus matrix. The range of window size is suggested as (9, 12), not (6, 9) the regular setting.

**Figure 14:**
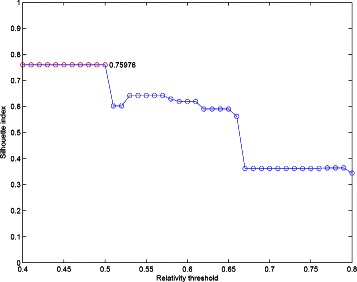
The clustering performance of our extended version of the proposed algorithm for the Yeast sporulation dataset.

To resolve the problem with short time-series gene expression datasets, Ernst et al. present an algorithm to analyses and retain significant gene expression profiles (Ernst et al. [35]). In this paper, we use our extended version of proposed algorithm for choosing more combinations from interval selection as the number of times to apply affinity propagation in order to increase the number of votes for investigating the relationship between genes. Our extended algorithm has a better value of the Silhouette index 0.75976 depicted in Figure 14.

Compare with the results of other methods including SiMM-TS [32], Chiu et al. [34], VGA [36], SOM [4], and CRC [37], our result shows better performance, which are shown in Table 3.

**Table 3:** **The comparison of performance for the Yeast sporulation dataset**

**Method**	**The Silhouette index**
Our Extended Version	0.75976
Our Algorithm (13 points)	0.72923
Chiu *et al.*’s [34]	0.63920
SiMM-TS [32]	0.62470
VGA [36]	0.57030
Average linkage	0.50070
SOM [4]	0.58450
CRC [37]	0.56220

In the implementation of the extended version of our algorithm, the execution time may rapidly increase because the number of combinations grows exponentially. Our suggestion is applied this version on those datasets with small size of time points, say 10 at most.

**Figure 15:**
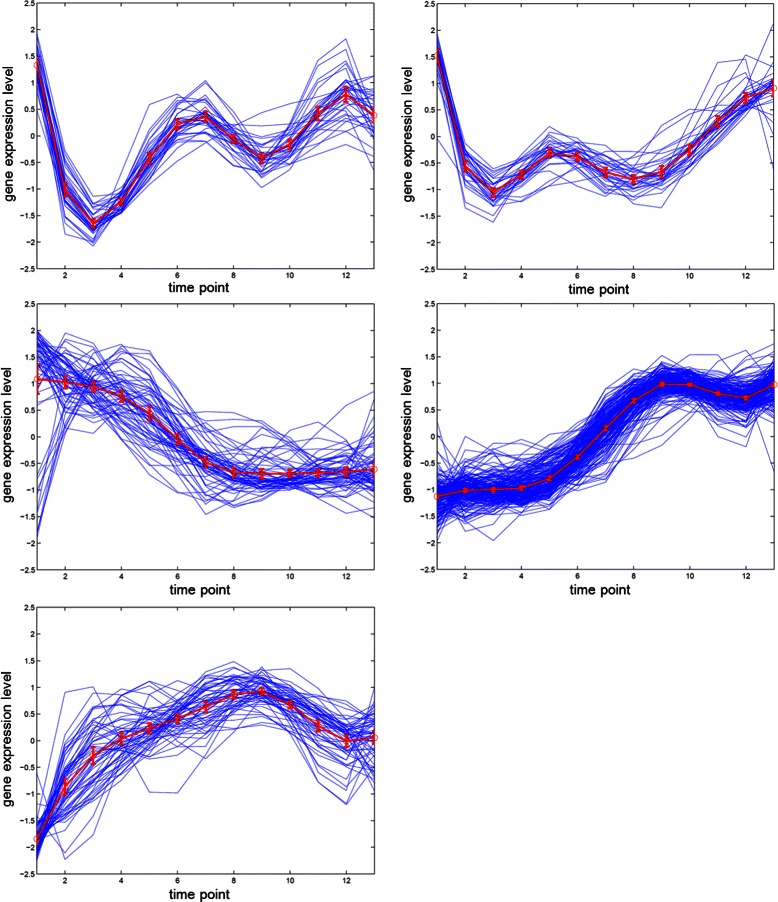
The gene expression results of our proposed algorithm for the Yeast sporulation dataset with 13 time points.

**Figure 16:**
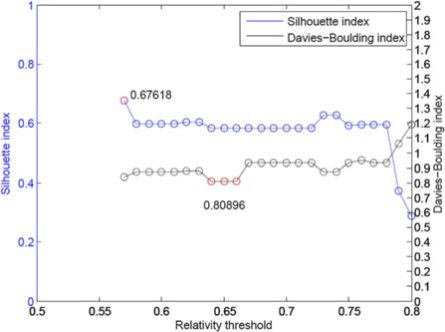
The clustering performance of our proposed algorithm for the Yeast sporulation dataset with the original 7 time points.

We demonstrate the clustering result with five groups derived from our algorithm in Figure 15. The clustering performance of our proposed algorithm for the Yeast sporulation dataset with the original 7 time points in Figure 16.

The statistics of the execution time for three dataset is shown in Table 4. A large amount of features extracted using our sliding-window mechanism can help to enhance the accuracy practically. Even though the affinity propagation is performed many times, the execution time of each dataset is appropriate. For the case of Y5 dataset, our interpolation version costs 177.851 seconds; the interpolation version of the Yeast sporulation dataset of 13 points costs 32.803 seconds; and for the Yeast galactose dataset, the execution time is 27.243 seconds. Note that the maximum number of iterations is 1000, if the AP procedure cannot converge.

**Table 4:** **Execution time and the number of genes for the proposed algorithm on three different datasets**

**Dataset**	**Exec. time**	**# of genes**	**Points**	**# of votes**
Y5	177.851	384	33	126
Sporulation	32.803	474	13	14
Galactose	27.243	205	20	51

## Conclusion

In this paper, we present an unsupervised clustering algorithm to analyze time-series gene expression data, which requires no prior knowledge, such as the number of clusters or the cluster exemplars (centroids). The algorithm combines affinity propagation and consensus clustering with various intervals of time points, which provided progressive robustness and accuracy by overcoming the interference from background noises and experimental errors. Besides, the interactions between genes across distinct time points were investigated by interval selection based on a sliding-window mechanism.

Because of the efficiency of affinity propagation, the proposed algorithm provides appropriate and effective analyses of time-series gene expression data. Based on three real gene expression datasets, our algorithm significantly outperformed other methods when the same datasets were used in the evaluation. The experimental results on the Yeast galactose dataset, the Yeast cell-cycle dataset (Y5), and the Yeast sporulation dataset, confirmed that our method can successfully illustrate biological relevance between the expressed genes.

In the future development of our method, we aim to integrate the problem of absent features at some time points, which is a critical issue in bioinformatics and machine learning. The standard treatments for absent features such as Zero (missing values set to zero), Mean (set to the mean value of the feature over all the data), and kNN (set with the average value obtained from the K nearest neighbors) in time-series data neglect the temporal dependence, causing improper results. We also aim to improve the semi-supervised clustering analyses, which are currently affected by the incompleteness of the gene annotations. By combining un-annotated data with the small amount of annotated data that is available, we expect to see considerable improvements in the clustering accuracy of our method.

## Availability

The program is freely available for non-profit use on request at https://vc.cs.nthu.edu.tw/tychiu/Programs/MainProgram.7z.
